# *Aedes* species (Diptera: Culicidae) ecological and host feeding patterns in the north-eastern parts of South Africa, 2014–2018

**DOI:** 10.1186/s13071-021-04845-9

**Published:** 2021-06-26

**Authors:** M. M. Guarido, M. A. Riddin, T. Johnson, L. E. O. Braack, M. Schrama, E. E. Gorsich, B. D. Brooke, A. P. G. Almeida, Marietjie Venter

**Affiliations:** 1 Zoonotic Arbo- and Respiratory Virus Program, Department Medical Virology, Faculty of Health Sciences, Centre for Viral Zoonoses, University of Pretoria, Pathology Building, Prinshof Campus South, Private Bag X323, Gezina, Pretoria, 0031 South Africa; 2 Present Address: Faculty of Health Sciences, UP Institute for Sustainable Malaria Control, University of Pretoria, Pretoria, South Africa; 3 Institute of Environmental Sciences, Leiden University, Leiden, The Netherlands; 4 School of Life Sciences, University of Warwick, Coventry, UK; 5 The Zeeman Institute for Systems Biology and Infectious Disease Epidemiology Research, University of Warwick, Coventry, UK; 6 Centre for Emerging Zoonotic and Parasitic Diseases, National Institute for Communicable Diseases/NHLS, Johannesburg, South Africa; 7 Wits Research Institute for Malaria, School of Pathology, University of the Witwatersrand, Johannesburg, South Africa; 8 Institute of Tropical Medicine and Hygiene (IHMTNOVA), Medical Parasitology Unit/GHTM, NOVA University of Lisbon, Lisbon, Portugal; 9 Malaria Consortium, Faculty of Tropical Medicine, Mahidol University, Bangkok, Thailand; 10 Present Address: Department of Biological Sciences, Copperbelt University, Kitwe, Zambia

**Keywords:** *Aedes*, Mosquitoes, Disease vectors, Africa, South Africa

## Abstract

**Background:**

There is a paucity of recent data and knowledge on mosquito diversity and potential vectors of arboviruses in South Africa, with most of the available data dating back to the 1950s–1970s. *Aedes* and *Culex* species are the major vectors of some of the principal arboviruses which have emerged and re-emerged in the past few decades.

**Methods:**

In this study we used entomological surveillance in selected areas in the north-eastern parts of South Africa from 2014 to 2018 to assess mosquito diversity, with special emphasis on the *Aedes* species. The impact of trap types and environmental conditions was also investigated. Identification of the blood meal sources of engorged females collected during the study period was carried out, and DNA barcodes were generated for selected species.

**Results:**

Overall, 18.5% of the total *Culicidae* mosquitoes collected belonged to the genus *Aedes*, with 14 species recognised or suspected vectors of arboviruses. Species belonging to the *Neomelaniconion* subgenus were commonly collected in the Bushveld savanna at conservation areas, especially *Aedes mcintoshi* and *Aedes circumluteolus*. *Aedes aegypti* was present in all sites, albeit in low numbers. Temperature was a limiting factor for the *Aedes* population, and they were almost exclusively collected at temperatures between 18 °C and 27 °C. The cytochrome oxidase subunit I (COI) barcode fragment was amplified for 21 *Aedes* species, and for nine of these species it was the first sequence information uploaded on GenBank.

**Conclusion:**

This study provides a better understanding of the diversity and relative abundance of *Aedes* species in the north-east of South Africa. The information provided here will contribute to future arboviral research and implementation of efficient vector control and prevention strategies.

**Graphical abstract:**

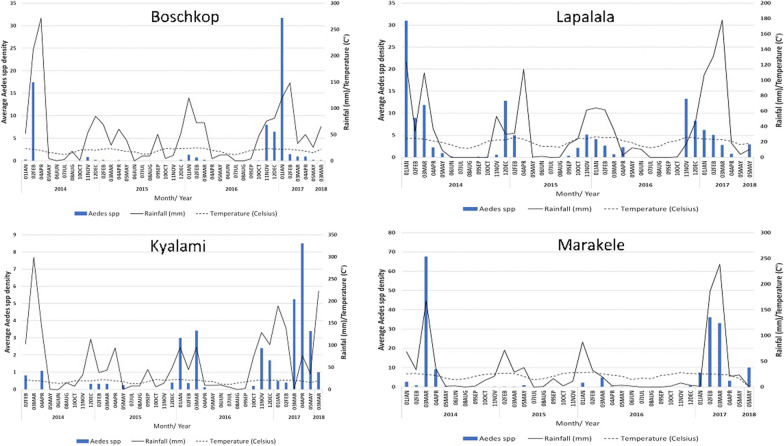

**Supplementary Information:**

The online version contains supplementary material available at 10.1186/s13071-021-04845-9.

## Background

Multiple mosquito species transmit pathogens of medical and veterinary importance to humans and animals, and mosquitoes are therefore considered one of the most important arthropod groups in the public health field [1, 2].

Vector-borne diseases are being reported with greater frequency due to the global movement of humans, animals, and goods, in combination with climate change [3] and the impact of land use and urbanisation [4]; this is especially evident with respect to *Aedes*-related arboviruses. Information such as the distribution, abundance, and seasonality of vectors, combined with an understanding of their relationship with the environment, is required to develop and implement successful vector control programs [5].

Knowledge of vector host preferences is important for understanding arboviral circulation between vectors, animals, and humans. The feeding behaviour is affected by intrinsic and extrinsic factors [6], and host selection by mosquitoes can be opportunistic and affected by the presence and/or abundance of the various vertebrate species [7].

Another important step for effective monitoring of vectors is the correct identification of the species. Morphological identification uses published keys and relies on external features of adult mosquitoes. For that, the specimens must be handled and stored carefully, a process which is time-consuming and requires specific expertise. Moreover, it does not identify genetic variations and phenotypic plasticity which can be manifested at the species level and in vector competence [8]. DNA barcoding is becoming more popular as an efficient methodology to complement morphological taxonomy. However, available studies using DNA barcoding in southern Africa are mostly focused on Anophelinae mosquitoes [9, 10].

Historically, a large number of studies were published in South Africa during the 1950s and 1970s associated with outbreaks in livestock and in regional surveys on arboviruses affecting humans [11–14]. Recently, Cornel et al. [15] investigated the diversity and abundance of mosquitoes in southern Africa, and Gorsich et al. [16] compared different trapping methodologies to assess adult mosquito populations in South Africa. However, there is a paucity of recent data about the diversity, density, seasonality, biology, and molecular identification of mosquito species, especially for *Culicinae* mosquitoes, and *Aedes* species in particular. The landscape of most of southern Africa has been altered as a result of human activity. Recent data are helpful for understanding how mosquito diversity has changed since the 1950s–1970s and how the risk of arboviral circulation differs between humans and animals.

The aim of this study was to assess the broad patterns of *Aedes* mosquito species diversity and abundance in different habitats across selected sites in the north-eastern parts of South Africa. Additionally, the influence of climatic features in the faunistic composition of *Aedes* species within each region was analysed and the relative efficiency of different trap types was compared. The blood meals from engorged females collected during the study period were also determined using molecular methods, and DNA barcodes were sequenced to identify and characterise *Aedes* species in South Africa. The collected data will assist with risk assessment for disease outbreaks in the region.

## Methods

### Study areas

**Fig. 1:**
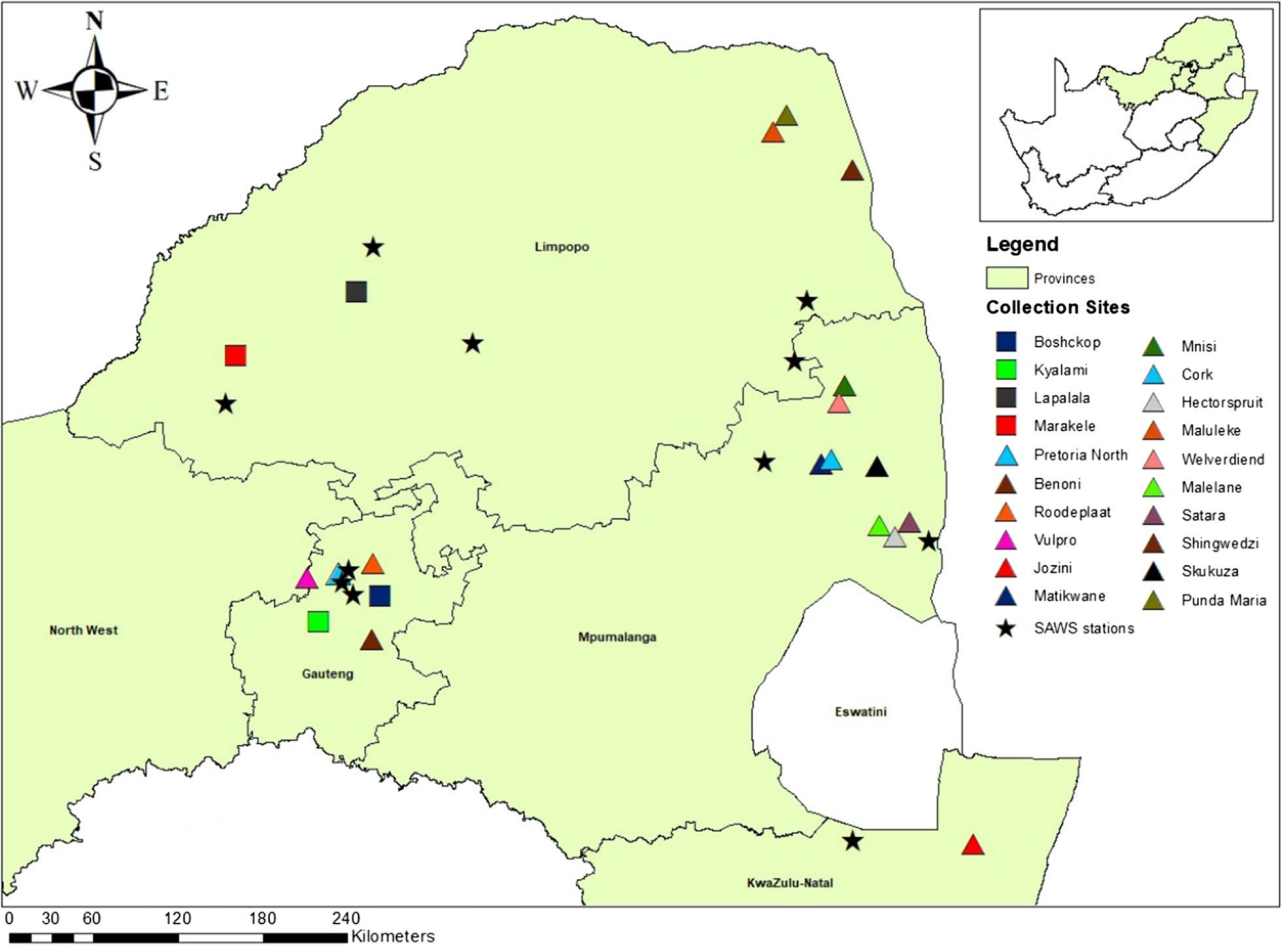
Mosquito collection sites and SAWS stations, South Africa, surveyed from January 2014 to May 2018. Squares indicate sentinel sites, triangles indicate ad hoc sites, and stars indicate SAWS stations

The study was conducted in five provinces of South Africa, namely, Gauteng, Limpopo, North West, Mpumalanga, and KwaZulu-Natal (Fig. 1). Mosquitoes were sampled monthly from January 2014 to May 2017 from sentinel sites and from supplementary collections in ad hoc sites following the detection of arboviral occurrence in animal or human hosts. Additional collections were performed from March to April 2017 in and around Kruger National Park (KNP) [16, 17], and mosquitoes caught in this sampling were used only for molecular identification and blood meal analyses. In 2018 only one collection per site was performed from January to May. Conservation areas were sampled in order to collect sylvatic species in those areas where there is less interference from humans and livestock. Sylvatic species can also be important as arboviral vectors among wildlife species. Peri-urban areas were sampled because they are located between the urban and usually more rural farmland areas or between the urban and conservation areas. The ecological features associated with the sites sampled are Highveld (regions vary between 1200 and 1800 m in elevation), Middleveld Bushveld (varying between 600 and 1200 m), and Lowveld Bushveld (varying between 150 and 600 m). A summary of the collections per site are given in Table 1.

**Table 1:** Number of nights, trapping events, traps set, and mosquitoes collected per genera in the sentinel sites from January 2014 to June 2018

Sites	Trap events	Nights	Trap type^a^	N *An*	N *Ae*	N *Cx*	N *Ma*	N Other genera	Total Mosq.
Sentinel sites									
Peri-urban									
Boschkop	353	76	1, 2, 3	294	664	3153	1	1	4113
Kyalami	338	75	1, 2, 3	1793	577	6314	3	49	8736
Conservation									
Marakele	487	92	1, 2, 3	3213	2939	5234	166	108	11,660
Lapalala	568	98	1, 2, 3	9484	2522	2468	477	1724	16,675
Ad hoc sites									
Urban									
Pretoria North	23	7	2, 3	4	217	49	0	0	270
Matikwane	9	2	1, 2, 3	72	345	194	0	4	615
Peri-urban									
Benoni	31	5	1, 2, 3	165	348	677	0	1	1191
Roodeplaat	15	2	1, 2, 3	42	103	118	26	9	298
Vulpro	12	2	1, 2, 3	0	342	148	0	0	490
Rural									
Mnisi	171	45	1, 2, 3	821	864	2309	1042	9	5045
Jozini	47	12	2, 3	4332	2348	2229	2041	4	10,954
Hectorspruit	9	3	1, 2, 3	97	285	904	159	25	1470
Cork	9	3	1, 2, 3	2	19	35	0	3	59
Maluleke	9	3	1, 2, 3	20	37	148	11	5	221
Welverdiend	9	3	1, 2, 3	71	16	286	2	0	375
Conservation									
KNP Shingwedzi	25	5	1, 2, 3	693	118	336	15	9	1171
KNP Skukuza	27	5	1, 2, 3	235	53	190	40	1	519
KNP Satara	9	3	1, 2, 3	34	172	86	2	1	295
KNP Malelane	9	3	1, 2, 3	99	65	167	1	0	332
KNP Punda Maria	9	3	1, 2, 3	23	3	86	1	1	114
Total	2169	3		21,494	12,037	25,131	3987	1954	64,603

^a^1 = Mosquito net trap, 2 = BG-Sentinel trap, 3 = Centers for Disease Control and Prevention miniature light traps

*An*. *Anopheles*, *Ae*. *Aedes*, *Cx*. *Culex*, *Ma*. *Mansonia*, *N* total number collected, *Mosq*. mosquito, *KNP* Kruger National Park

Sampling was performed in different land use types: urban, peri-urban (horse farms), rural, and conservation areas (Additional file 1: Table S1). Trapping was carried out from 15:30–16:00 to 5:00–8:00, and sampling was conducted for 1–3 consecutive nights per site using multiple types of carbon dioxide (CO_2_)-baited traps: mosquito net, CDC miniature light traps (Centers for Disease Control and Prevention, USA), and BG-Sentinel traps (Biogents AG, Regensburg, Germany). Mosquitoes were collected from the net traps using aspirators and transferred to mesh-topped polystyrene cups. CDC miniature light traps were hung at least 1.5 m from the ground, baited with CO_2_. BG-Sentinel traps were added from 2017 and were additionally baited with a non-toxic lure. All the traps were placed at least 80 m apart to reduce trap interference.

Collected mosquitoes were immediately euthanised by freezing and were morphologically identified to species using published keys and descriptions [18–21]. Engorged females were separated by species, collection site, and date, and individually preserved at −80 °C until further analysis. Representative specimens were pinned as reference material, and 1–3 legs were individually preserved for further molecular work. The classification of Wilkerson et al. [22] for Aedini mosquitoes was adopted. Morphologically similar or indistinct species were recorded as belonging to a group as follows: *Aedes dentatus*, *Aedes leesoni*, *Aedes dentatus/leesoni*, or *Aedes vexans*. *Aedes* specimens that were too badly damaged to identify morphologically to species were identified to the genus or in a species group following the groups mentioned above. Some adult female species were not distinguishable morphologically and these were recorded as either of the two possible species they could be. The pinned reference material was also compared with the specimens deposited at the National Institute for Communicable Diseases of South Africa (NICD).

### Climatic conditions

Climate data were obtained from 11 permanent stations of the South African Weather Service (SAWS) closest to the sampling sites [23] (Fig. 1). Data obtained from SAWS included total hourly rainfall, hourly average wind speed, hourly average air temperature, and hourly average humidity. For this analysis, calculations for the climatic variables were as follows: air temperature (°C) (average daily, average nightly from 16:00 to 7:00, average prior 48 h, average prior 15 days, average prior 30 days), rainfall (mm) (total daily, total nightly from 16:00 to 7:00, total prior 48 h, total prior 15 days, total prior 30 days), wind speed (m/s) (average daily, average nightly from 16:00 to 7:00, average prior 48 h), and humidity (%) (average daily, average nightly from 16:00 to 7:00, average prior 48 h, average prior 15 days, average prior 30 days).

### DNA extraction, DNA barcoding, and blood meal analyses

DNA was extracted using the DNeasy^®^ Blood & Tissue Kit (Qiagen, Valencia, CA, USA), according to the manufacturer’s guidelines. DNA from the leg (1 up to 3 legs) of each mosquito and DNA from freshly engorged females was extracted.

The barcode region of mtDNA of subunit I of the cytochrome oxidase (COI) gene was amplified using universal primers [24]. The 50 µl polymerase chain reaction (PCR) consisted of 5 μl of the extracted DNA, 1 μl of 10 mM deoxynucleotide triphosphates (dNTPs), 10 μl of buffer, 0.5 μl Phusion^®^ High-Fidelity DNA Polymerase (Thermo Fisher Scientific™), and 1 μl of 20 μM of each primer. PCR conditions were as follow: 98 °C for 30 s followed by 35 cycles of 98 °C for 30 s, 52 °C for 45 s, and 72 °C for 30 s, with a final extension of 72 °C for 5 min using a thermocycler (Applied Biosystems™).

Molecular identification of the blood meal was performed targeting cytochrome b (CYTB). Published primers targeting mammal [16, 17] and avian [16, 18] genetic markers were selected for amplification, and combined with Phusion^®^ High-Fidelity DNA Polymerase (Thermo Fisher Scientific™). The PCR was performed in a final volume of 50 µl which consisted of 20 µM of each primer, 10 mM dNTPs, and 10 µl DNA. The reaction mix was then subjected to an initial incubation of 95 °C for 5 min followed by 36 cycles of 94 °C for 30 s, 55 °C (mammal) or 60 °C (avian) for 45 s, and 72 °C for 90 s, with a final extension of 72 °C for 7 min using a thermocycler (Applied Biosystems™).

### Gel electrophoresis and sequencing

PCR products were viewed by 2.0% agarose gel electrophoresis containing ethidium bromide. Amplicons of the correct size were excised from the gel and purified using a Zymoclean™ Gel DNA Recovery Kit (Zymo Research, CA, USA) according to the manufacturer’s instructions. Purified amplicons were bidirectionally sequenced using the BigDye^®^ Direct Cycle Sequencing Kit (Thermo Fisher Scientific™, MA, USA) and sent to the University of Pretoria DNA sequencing facility or Inqaba Biotec™ (Pretoria, South Africa) for Sanger sequencing.

### Data analysis and phylogenetic analysis

Analyses and interpretation were performed separately for sentinel collections and ad hoc sites due to the variation in the number and method of collection events. Individual site adult mosquito densities were calculated as mean mosquitoes per trap-night by dividing the total collection at each site by the number of collection nights and number of traps utilised. There were 21 trap failure events as a result of battery failure or equipment damage from environmental factors or animals, and these collections were excluded prior to analysis.

Species richness and species diversity were calculated for each site and for each trap type. Species richness is reported as the number of mosquito species collected at each site. Species diversity was estimated by calculating the Simpson diversity index. The presumption for normality was tested using the Shapiro–Wilk test. To investigate whether there were significant differences between *Aedes* species composition within trap types, we applied the Wilcoxon rank-sum test (*n* = 2, two independent variables) or Kruskal–Wallis *H* tests (*n* > 2, three or more independent variables). Significant values were adjusted by the Bonferroni correction for multiple tests. The trap analyses were performed using data from collections at sentinel and ad hoc sites in 2017 and 2018. CDC light traps were considered the same trap type independently of the type of light used. The effect of environmental factors was tested using Spearman correlation analysis to examine the relation between the different variables and log_10_ abundance of *Aedes* mosquitoes per trap-night. Regression analysis was also performed to clarify the relationship and *R*-squared values were calculated. Differences were considered statistically significant at a value of *p* ≤ 0.05. Species richness and species diversity were calculated using the ‘entropart’ [25] package in R version 3.6.1 [26]. All statistical analyses were completed using IBM SPSS version 26 software (IBM Corp., Armonk, NY, USA). Graphics were generated using Microsoft Excel (version 2010) [27].

The resulting sequences were edited and analysed using the QIAGEN CLC Main Workbench version 8.0.1 [28]. Sequences were compared with the databases available in GenBank and the Barcode of Life Data System (BOLD) accordingly with the gene fragment, using the Basic Local Alignment Search Tool (BLAST). Multiple sequence alignments were compiled using the online version of Multiple Alignment using Fast Fourier Transform (MAFFT) with default parameters. The model of best fit was identified, and a maximum likelihood tree was produced using Molecular Evolutionary Genetics Analysis (MEGA) version 7.0 [29]. For barcode analyses, sequence statistics and pairwise sequence divergence were calculated based on the Kimura 2-parameter (K2P) model.

## Results

### Mosquito collection

A total of 64,603 adult mosquitoes belonging to 11 genera were collected across sentinel and ad hoc sites. The most common genus collected was *Culex* (38.90%, *N* = 25,131), followed by *Anopheles* (33.27%, *N* = 21,494), *Aedes* (18.63%, *N* = 12,037), *Mansonia* (6.17%, *N* = 3987), and other genera combined (3.03%, *N* = 1954, *Uranotaenia*, *Aedeomyia*, *Ficalbia*, *Coquillettidia*, *Mimomyia*, *Culiseta*, and *Eretmapodites*). *Culex*, *Aedes*, *Anopheles*, and *Mansonia* were the most important genera collected with respect to medical and veterinary significance. A total of 12,037 adult mosquitoes belonging to the *Aedes* genus were collected in all the sites from January 2014 to May 2018 (Table 1). At the sentinel sites, a total of 6702 aedine species were collected (10.37% of the total *Culicidae* collected), while a total of 5335 (8.26% of the total) were collected at the ad hoc sites.

While the overall number of *Aedes* mosquitoes collected in Kyalami and Boschkop was relatively low, these two sites had the highest biological diversity index. On average, species diversity was highest in Kyalami (Simpson’s Diversity Index 1− *D* = 0.88), followed by Boschkop (0.78), Lapalala (0.74), and Marakele (0.30) (Additional file 1: Table S2). Lapalala had the highest *Aedes* species richness, but it was not consistently the most biologically diverse site. This is most likely because of *Aedes* (*Neomelaniconion*) *mcintoshi* (42.62%) comprising a large proportion of the collection from this site, thus skewing the diversity index due to the abundance of one species. A similar result was observed in Marakele: even though the species richness was high, a large proportion of the collection were from *Ae. mcintoshi* (82.81%), making the biological diversity index exceptionally low (0.31).

For the ad hoc sites, Mnisi (25 species from 937 *Aedes* mosquitoes identified) and Jozini (17 species from 2348 *Aedes* mosquitoes identified) had the highest *Aedes* species richness. *Aedes mcintoshi* (45.99%, *n* = 431) and *Aedes* (*Stegomyia*) *aegypti* (22.30%, *n* = 209) were the most common species in Mnisi. Jozini exhibited a dominant species of *Aedes* (*Aedimorphus*) *durbanensis* (66.26%, *n* = 1556). The species diversity indexes were highest in KNP Shingwedzi (Simpson’s Diversity Index = 0.82), followed by Mnisi (0.70), KNP Skukuza (0.65), Roodeplaat (0.64), Vulpro (0.62), Benoni (0.52), Jozini (0.51), Matikwane (0.32), and Pretoria North (0.07) (Additional file 1: Table S3).

In this survey, 14 *Aedes* species which are recognised or suspected vectors of mosquito-borne viruses in southern Africa were collected. The most abundant potential vector collected throughout the study was the floodwater species *Ae. mcintoshi*, which occurred in high numbers in Middleveld locations such as Marakele and Lapalala (Limpopo Province at conservation sites) (Additional file 1: Fig. S1). *Aedes fowleri* and *Ae. vittatus* also appeared in high numbers specifically in Lapalala. *Aedes aegypti* occurred in all sentinel sites but in low numbers. Species from the *dentatus* group including *Ae. dentatus*, *Ae. cumminsii*, and *Ae. pachyurus*, the *leesoni* group, *Ae. juppi*, and *Ae. unidentatus* were common in the Highveld (Gauteng province at peri-urban sites) (Additional file 1: Fig. S1).

### Effect of trap type on *Aedes* spp. abundance

There was a significant difference between the abundance of *Aedes* mosquitoes captured by different traps in sentinel sites (Kruskal–Wallis test: *p*-value < 0.05, Additional file 1: Table S4). In Marakele and Lapalala there was a significant difference in aedine captured between BG-Sentinel traps and net traps (Wilcoxon rank-sum test, *V* = 18.5—Marakele and *V* = 0—Lapalala, *p* < 0.05), and in Boschkop we found a significant difference between BG-Sentinel trap and CDC light traps (CDC-LT) (Wilcoxon rank-sum test, *V* = 10, *p* < 0.05). Net traps exhibited the highest *Aedes* species richness, with 24 species collected, followed by CDC-LT with 22 and BG-Sentinel with 20 aedine identified. There were two species that were collected exclusively in BG-Sentinel traps (*Ae. vexans* group and *Aedes breedensis*), one species only found in net traps (*Aedes mixtus/microstictus*), and one species found only in CDC-LT (*Aedes aerarius*). The *Aedes* species diversity index was highest at the CDC-LT (Simpson’s Diversity Index = 0.83), followed by BG-Sentinel (0.75) and net traps (0.56).

The ad hoc site collections yielded no significant “between-trap” difference in *Aedes* species captured (Kruskal–Wallis test: *p* = 0.376). The two sites with the largest *Aedes* collection, Mnisi and Jozini, showed a significant difference between BG-Sentinel and CDC-LT (Wilcoxon rank-sum test, *V* = 12, *p* < 0.05), and BG-Sentinel and net traps (Wilcoxon rank-sum test, *V* = 36, *p* < 0.05) at Mnisi, and a significant difference between BG-Sentinel and CDC traps (the only trap type used at this site) (Wilcoxon rank-sum test, *V* = 17, *p* < 0.05) in Jozini.

Overall, looking at the most abundant recognised or potential vectors collected, *Ae. aegypti* was predominantly collected in BG-Sentinel traps (56.87%, *n* = 484) and *Ae. mcintoshi* in net traps (93.01%, *n* = 1278).

### Climatic conditions

**Fig. 2:**
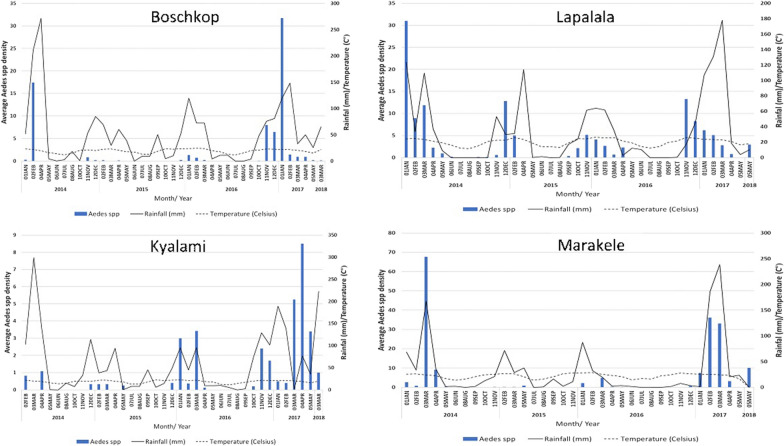
*Aedes* mosquito abundance per trap-night from January 2014 to June 2018 at the sentinel sites

The population structure and size of *Aedes* species fluctuated with season during the study period (Fig. 2). An overall trend was evident in the mean number of *Aedes* mosquitoes per trap-night, which was lower in the drier months (June to October) than during the wetter months (November to mid-April). The population peaks appeared to correlate with rainfall events and the highest mean temperatures. *Aedes mcintoshi*, the most abundant aedine within the study, exhibited higher population numbers during the rainy season. The population peaked in January to March 2014, and during the same period in 2017, coinciding with the highest rainfall (Additional file 1: Fig. S2). *Aedes* (*Fredwardsius*) *vittatus* appeared shortly after the first episodes of rain each year, and their population increased from November to January (Additional file 1: Fig. S2). Interestingly, *Aedes fowleri* mosquitoes were only collected in the early wet season (November and December) of 2016 and not in the other years.

A positive correlation was observed between the abundance of *Aedes* per trap-night and the environmental conditions including average temperature 30 days prior to collection, average rainfall 30 days prior to the collection event, and average humidity 15 days prior to collection, with Spearman rank correlations of 0.533 (*p* < 0.001), 0.498 (*p* < 0.001), and 0.388 (*p* < 0.001), respectively (Additional file 1: Table S5). There was no significant correlation with average daily rainfall. The effect of wind on breeding, feeding, and flight was also considered as an important factor, particularly during the collection event. However, there was a negative correlation between the abundance of *Aedes* per trap-night and wind variables tested (Additional file 1: Table S3). The effect of air temperature on the abundance of aedine mosquitoes showed a relationship with aedine abundance during temperatures between 18 °C and 27 °C. The regression relationship (*R*^2^) between the abundance of *Aedes* per trap-night and average temperature 30 and 15 days prior to collection, average rainfall 30 and 15 days prior to the collection event, and average humidity 30 and 15 prior to collection was quite low, ranging between 0.031 and 0.157, respectively, which is shown by a scatter plot (Additional file 1: Fig. S3).

### Molecular identification

A total of a 52 COI sequences were generated in this study from 21 *Aedes* species belonging to nine subgenera: *Stegomyia* (4 *Ae. aegypti*, 2 *Ae. simpsoni*, 1 *Ae. ledgeri*), *Aedimorphus* (1 *Ae. hirsutus*, 2 *Ae. vexans*, 3 *Ae. fowleri*, 4 *Ae. cumminsii*, 1 *Ae. pachyurus*, 5 *Ae. eritreae*, 3 *Ae. durbanensis,* 1 *Ae. quasiunivittatus*), *Catageiomyia* (5 *Ae. microstictus*), *Neomelaniconion* (2 *Ae. aurovenatus*, 4 *Ae. mcintoshi*, 1 *Ae. circumluteolus*, 3 *Ae. unidentatus*), *Fredwardsius* (2 *Ae. vittatus*), *Ochlerotatus* (3 *Ae. juppi*), *Mucidus* (2 *Ae. sudanensis*), *Albuginosus* (2 *Ae. haworthi*), and *Diceromyia* (1 *Ae. furcifer*).

**Fig. 3:**
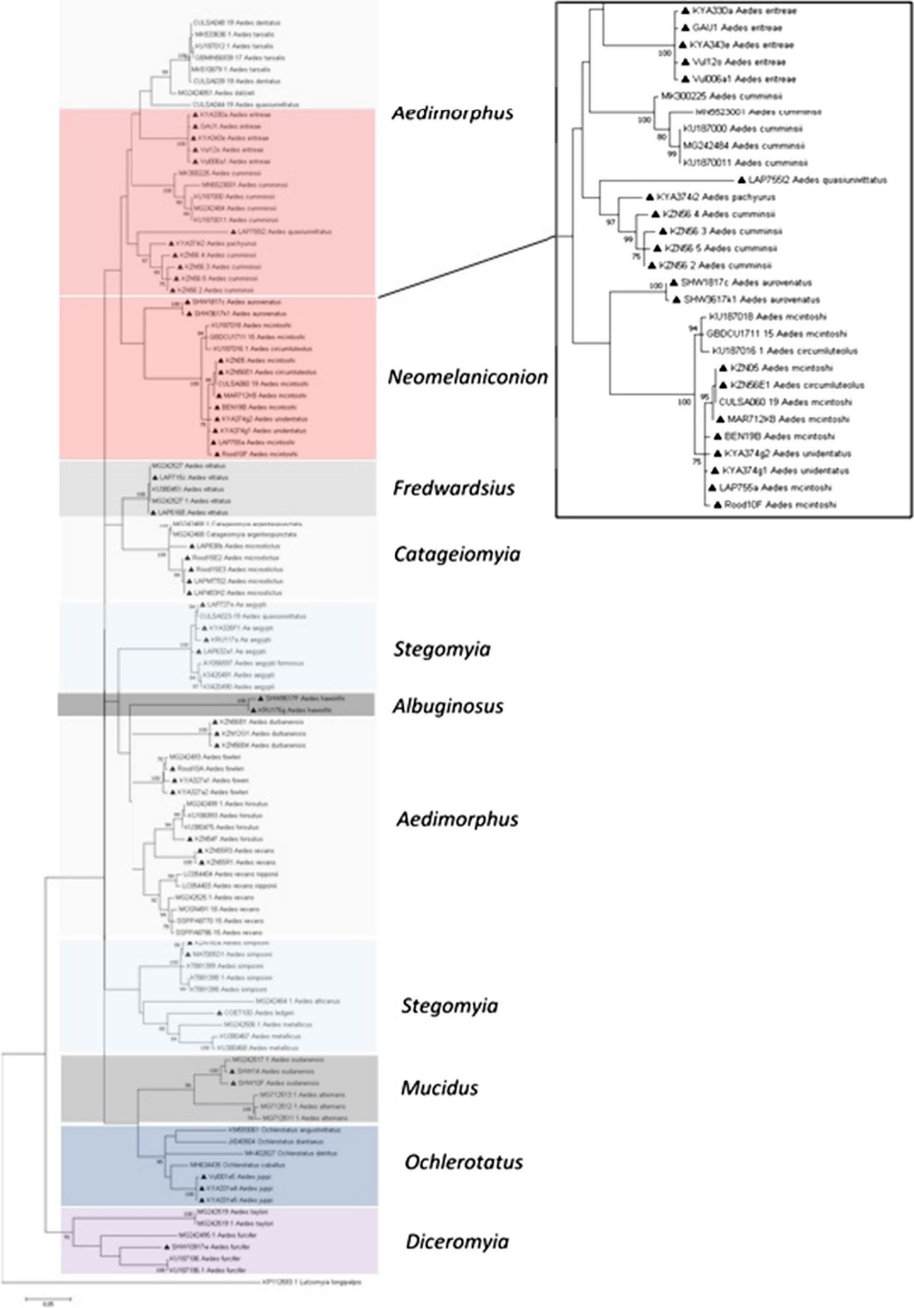
Phylogenetic tree of sequences from *Aedes* mosquitoes based on 111 sequences and 507 base pairs of COI gene. The tree was constructed with MEGA 7, using the maximum likelihood method and the Tamura-Nei model with 1000 bootstrap replicates. The tree with the highest log likelihood (−6035.72) is shown. GenBank accession numbers are indicated. Numbers on internal branches indicate bootstrap values. Samples which are part of this study are marked with a black triangle

DNA barcode sequences consisting of 507 bp produced in this study and sequences retrieved from NCBI GenBank and BOLD reported from Africa and worldwide were aligned and used to build a maximum likelihood tree (Fig. 3) as confirmation of morphological mosquito identification. The results show the evolutionary distances using the Tamura-Nei model with a bootstrap tested with 1000 replicates (30). The phylogenetic tree of COI showed *Lutzomyia longipalpis* species separated as outgroup from the aedine tested. The rest of the aedine taxa were divided into two clusters: one was a large cluster, and the other was a well-supported cluster containing only isolates belonging to the subgenus *Mucidus* (*Ae. sudanensis* and *Aedes alternans*). The larger cluster was further divided into a small cluster comprising *Ochlerotatus* species and a larger cluster of the remaining subgenera. Species belonging to *Aedimorphus* subgenera were recovered in different clusters, suggesting that this subgenus is not monophyletic in this study. Sequences produced here from mosquitoes which were identified morphologically as *Ae. cumminsii* (Additional file 1: Fig. S4) clustered with *Ae. pachyurus* (of the same group) and *Ae. quasiunivittatus* (of another group within the same subgenus) and did not cluster together with sequences from *Ae. cumminsii* from Kenya (KU187000.1, KU187001.1, and MK300225), Guinea (MN552300.1), or Senegal (MG242484). Species belonging to the *Neomelaniconion* subgenus were divided into two clusters: a small one containing only *Ae. aurovenatus*, and a larger one with no separation between *Ae. unidentatus*, *Ae. mcintoshi*, and *Ae. circumluteolus*.

The sequences analysed showed a nucleotide diversity of 0.097. Total nucleotide composition of the COI fragment varied slightly across the tested samples, and the sequences were highly adenine/thymine (AT)-rich, ranging from 66.27 to 71.40%, while guanine/cytosine (GC) content ranged from 28.60 to 33.73%. The distance matrix calculated for the COI sequence for all the samples showed an overall K2P average distance of 0.105. Data analysis for sequence divergence revealed intraspecies divergence ranging from 0.0 to 2.83%; the higher value was used as a cut-off to define the limit for species identification, and interspecies divergence ranged from 3.24 to 16.27%. The highest genetic distance was for *Ae. sudanensis* collected in KNP Shingwedzi (from 9.91 to 15.29%) to the other samples. The K2P interspecific distance between the members of *Neomelaniconion* subgenera ranged from 0.20 to 9.92%, with the highest genetic distance between *Aedes aurovenatus* (from 8.12 to 9.92%) and the other members of *Neomelaniconion* species. The K2P interspecific distance ranged from 0.20 to 1.20% between *Ae. mcintoshi*, *Ae. circumluteolus*, and *Ae. unidentatus.*

### Blood meal analyses

Engorged *Aedes* females comprised < 1% of the adults collected. That was expected due to characteristics of the trap methodology used for collection, which is more attractive to host-seeking adult females and not to recently blood-fed individuals. Despite this trap limitation, 112 freshly blood fed-females were caught between January 2014 and May 2018. The specimens tested represented 16 *Aedes* species. Sampling conducted in Kruger National Park (Shingwedzi area) and Marakele National Park had the greatest number of engorged females (*n* = 19/112 per site, 16.96%), followed by Lapalala (*n* = 18/112, 16.07%). Of the individuals tested, 64/112 (54.46%) were successfully identified for blood meal origin, which showed 62/64 (96.88%) mammal and 2/64 (3.12%) avian host species (Table 2).

**Table 2:** *Aedes* species, collection site, and host identified through sequencing of partial cytochrome b gene

*Aedes* species	Location	Host blood meal
*Ae. aegypti*	Kyalami, KNP-SHI	Domestic cat (*Felis silvestris catus*), northern puffback (*Dryoscopus gambensis*)
*Ae. aurovenatus*	KNP-SHI	African buffalo (*Syncerus caffer*)
*Ae. circumluteolus*	Jozini	Cattle (*Bos taurus*)
*Ae. dentatus*	Kyalami	Horse (*Equus caballus*)
*Ae. dentatus* group	Benoni	Cattle (*Bos taurus*)
*Ae. durbanensis*	Jozini	Cattle (*Bos taurus*), goat (*Capra hircus*), sheep (*Ovis aries*)
*Ae. eritreae/karooensis*	Vulpro	Bushbuck (*Tragelaphus scriptus*), nyala (*Tragelaphus angasii*), human (*Homo sapiens*)
*Ae. fowleri*	Lapalala	White rhinoceros (*Ceratotherium simum*), impala (*Aepyceros melampus*)
*Ae. hirsutus*	Marakele, KNP-Mal, KNP-Sat	White rhinoceros, blue wildebeest (*Connochaetes taurinus*), African buffalo, impala
*Ae. leesoni/alboventralis*	KNP-SHI	African buffalo, bushbuck, impala, kudu (*Tragelaphus strepsiceros*)
*Ae. mcintoshi*	Mnisi, Lapalala, Marakele, Vulpro	Cattle, hippopotamus (*Hippopotamus amphibius*), white rhinoceros, human, kudu, bushbuck, Cape vulture (*Gyps coprotheres*), blue wildebeest
*Ae. ochraceus*	KNP-Sat and KNP-SHI	African buffalo, impala
*Ae. pachyurus*	Benoni, Roodeplaat, Kyalami	Cattle, human, goat, common duiker (*Sylvicapra grimmia*), domestic dog (*Canis lupus*)
*Ae. quasiunivittatus*	Boschkop, Mnisi, Lapalala, KNP-Mal	Sheep, cattle, hippopotamus, white rhinoceros
*Ae. vittatus*	Mnisi, Lapalala	Cattle, human, waterbuck (*Kobus ellipsiprymnus*)
*Aedes* spp	Mnisi, Marakele	Impala

*Ae* *Aedes*, *KNP* Kruger National Park, *SHI* Shingwedzi, *Mal* Malelane, *Sat* Satara

The results revealed 16 different mammal species, of which buffalo (*Syncerus caffer*) was the predominant species detected (17%), followed by domestic cow (*Bos taurus*, 14%) and humans (*Homo sapiens*, 13%). Two different avian species, *Gyps coprotheres* (vulture) and *Dryocopus gambensis* (bushshrike), were detected. In species with multiple engorged females collected and successfully tested, host blood meal differed by up to five different hosts across the *Aedes* species (Additional file 1: Table S6).

## Discussion

To assess aedine mosquito community diversity in the north-eastern parts of South Africa, the present study collected adult mosquitoes based on adult trapping methods commonly used in southern Africa [15, 16, 31].

*Aedes* species were overall found in lower numbers compared to *Culex*, *Anopheles,* and other genera. In total, across all trap types and collection sites, 30 species belonging to the genus *Aedes* were collected. However, aedine species are considered generally day-biting mosquitoes, and as this study used traps to collect mosquitoes from sunset to sunrise, this would likely have contributed to bias against the capture of *Aedes* species. Larval sampling is an additional method that could further complement adult collection in future studies to understand the diversity of an *Aedes* community [32] but was not used in this study. The trap types used here were also known to be biased towards host-seeking females due to the use of CO_2_, which could justify the low numbers of relative freshly engorged females collected during the study period. A possible method that could be incorporated in future studies to increase the number of freshly blood-fed females collected would be to sample mosquitoes from resting sites [33, 34]. However, despite the low number of engorged females tested, a better understanding of host selection by *Aedes* species was possible. All examined specimens primarily or exclusively fed on mammals, which confirms prior literature on *Aedes* species’ host selection [20, 35, 36].

The comparison of the host preference suggests that *Aedes* are not specialist but rather more opportunistic mammophilic blood feeders. The specimens identified feeding on cattle were collected in rural (Mnisi and Jozini) or peri-urban (Benoni) areas. At rural sites, cattle were the primary livestock present, while in Benoni, a peri-urban area of small holdings outside of Johannesburg and Pretoria, there are many horses but also other animals such as cattle and domestic dogs. Consequently, it is not surprising that all the species found to feed on cattle were collected from these sites. Species that were identified feeding on goats and sheep were collected in sites localised in peri-urban areas (Boschkop and Kyalami) on horse farms where no cattle were present, although goats and sheep were commonly found. The *Aedes* species that were detected feeding on wildlife were mainly collected in conservation areas (Marakele, Lapalala, and KNP).

*Aedes mcintoshi/circumluteolus* was the most abundant aedine sampled in the conservation regions. They are recognised as floodwater species that feed on livestock such as sheep, goats, and cattle, but also feed on humans [20]. Here, *Ae. mcintoshi* exhibited the highest feeding diversity and was identified feeding on cattle, hippopotamus, white rhinoceros, humans, kudu, bushbuck, blue wildebeest, and vultures, while *Ae. circumluteolus* was identified feeding on cattle. The variety of blood-feeding hosts can be attributed to the composition and abundance/availability of the hosts in each collection site. Additionally, these species are known as potential arboviral vectors, particularly in the Bushveld savanna (Marakele and Lapalala), and they were abundantly collected in the net traps. The species are widely distributed in South Africa and have been collected in the Karoo, Highveld, Lowveld, and the coast of Kwazulu-Natal Province [11, 37, 38]. They play a major role in Rift Valley fever (RVF) occurrence in South Africa, principally in the Karoo and Highveld (1950s and 1970s), during years that experienced much higher than average rainfall [51]. In such conditions, *Ae. mcintoshi* appeared to be the most important vector in the inland regions, while *Ae. circumluteolus* were more infected with RVF virus in KwaZulu-Natal Province [39]. More recently, in 2010–2011, an epidemic occurred with more than 14,000 animal cases recorded in eight out of nine provinces in South Africa [40], and in 2018, RVF was detected in a single farm in the Free State Province [41]. Furthermore, beyond their role in the maintenance of RVF, other arboviruses have been isolated from wild-caught mosquitoes in South Africa, including Wesselsbron virus (WSLB) and Middelburg virus (MIDV) in *Ae. mcintoshi/luridus*, *Ae. circumluteolus*, and *Ae. juppi/caballus* [42, 43].

*Aedes aegypti* was detected in this study across all habitats sampled, although in lower numbers. In urban sites, Matikwane and Pretoria North, *Ae. aegypti* was the dominant species. In general, this species was predominantly collected in BG-Sentinel traps. This trap is baited with lure as an attractant and is considered the gold standard for collection of *Aedes* mosquitoes, especially *Stegomyia* species [44, 45]. *Aedes aegypti* is the main vector for the four most important arboviruses, dengue (DENV), Zika (ZIKV), chikungunya (CHIKV), and yellow fever (YFV) viruses [46–50]. This species in Africa breeds predominantly in the domestic environment and in the ancestral sylvatic habitat [51]. In South Africa, there is an occurrence of a rural non-anthropophilic population of *Ae. aegypti* [52], which could explain why conservation areas exhibited lower overall numbers in comparison to urban and peri-urban sites. Previous studies have shown that both forms occur in South Africa: a non-anthropophilic community and anthropophilic populations [52, 53]. Such a finding could explain why some arboviruses such as CHIKV do not spread over large areas in southern Africa and have remained in rural settings [54]*. Aedes aegypti* was found to feed on domestic cats and on avian species. In other parts of Africa, this species has been detected feeding on humans, monkeys, oxen, goats, cats, dogs, rats, and fowl [55].

*Aedes dentatus*/*leesoni* group specimens were mainly collected in the peri-urban Highveld sites in Gauteng Province. Females of this group are often morphologically indistinct, particularly when there is interbreeding or when damage occurs during collection. *Aedes dentatus* is abundant primarily in the Highveld and feeds readily on humans and larger domestic animals [56]. In this study, the *Ae. dentatus* group was found feeding on horses in Benoni, a peri-urban site where mosquito sampling was performed in horse farms. Rift Valley, Sindbis (SINV), MIDV, Spondweni (SPOV), and Shokwe (SHOV) viruses were previously isolated from mosquitoes from this group [57–61].

Significant variation was found in the abundance of *Aedes* caught over the course of this survey. The highest numbers were collected during the wet season, especially between November and mid-April. Many studies have shown the influence of temperature and rainfall on *Aedes* populations [62–64]. The rainy season in most of the northern provinces in southern Africa is from November to April [65]. An increase in rainfall promotes the occurrence of temporary pools, a favourable habitat for many floodwater *Aedes* species, which include some major arboviral species [15]. The results showed rainfall as being the most influential climatic variable on *Aedes* populations, as *Aedes mcintoshi*, *Ae. vittatus*, and *Ae. fowleri* peaked soon after the beginning of the rains. This is an expected result, as they are considered floodwater species, with flooding events as a trigger for egg hatching [66]. The apparent drier months during the period from mid-2014 to 2016 when compared to the wetter seasons in 2016/2017 show variations in rainfall within South Africa during the study period [67, 68]. Annual rainfall has been shown to be decreasing in the north-eastern parts of the country [67, 68], while air temperature has been increasing over the past 50 years [75]. In Limpopo Province and parts of northern Mpumalanga Province, the changes are evident, with annual mean rain days translating to nearly 16 days and the temperature increasing by approximately 1 °C over a period of 50 years (1960–2010) [68]. Climate change can alter the geographical distribution of vectors and diseases such as DENV or CHIKV [69–71]. Temperature can be a survival-limiting factor for distribution of *Aedes* species [72]. In our results, *Aedes* individuals were largely collected at temperatures between 18 °C and 27 °C. Under laboratory conditions, *Ae. aegypti* has optimal adult longevity at around 21 °C [72], and below 14–15 °C they demonstrate reduced mobility and struggle to take a blood meal [73, 74], which supports the temperature thresholds and collection numbers in this study. Humidity can play a further integral role in mosquito survival, as they desiccate rapidly in dry conditions, thereby decreasing the survival rates [75]. Wind speed can also influence collection events, as high winds affect the flight behaviour of mosquitoes, and a 3 km/h wind speed can significantly reduce the host-seeking flight behaviour of present populations [76]. Humidity and wind speed during the trapping events correlated strongly with the abundance of mosquitoes, supporting such an association with mosquito behaviour and collection.

The genomic sampling in mosquito diversity has been improving recently; however, only 0.8% of the 3556 species recognised by the Mosquito Taxonomic Inventory are represented in NCBI GenBank, and the majority of them belong to Anophelinae [77]. There is also a lack of sequences belonging to African *Aedes* species in NCBI GenBank and BOLD databases, and this study enabled the addition of COI sequences for nine species: *Ae. eritreae*, *Ae. pachyurus*, *Ae. ledgeri*, *Ae. juppi*, *Ae. microstictus*, *Ae. aurovenatus*, *Ae. unidentatus*, *Ae. durbanensis*, and *Ae. haworthi*. Most of these species were last recorded in the 1950s to 1970s. *Aedes eritreae* had not been registered since 1971 when McIntosh et al*.* collected it in a single locality in the former province of Transvaal [56]. That was also true for *Ae. pachyurus*, where this species had only been collected in the Cape Provinces [78] and KwaZulu-Natal Province [56].

The *Neomelaniconion* cluster included sequences generated from species of *Ae. mcintoshi*, *Ae. circumluteolus*, *Ae. unidentatus*, and *Ae. aurovenatus*. With the exception of *Ae. aurovenatus*, all species clustered together, indicating limitations of the COI gene as a marker to separate these species. The specimens are morphologically similar but can be distinguished based on unguis shapes and wing scale patterns. Remarkably, though, they were very similar genetically. Evidence from Kenya suggests that *Ae. mcintoshi* is a complex of species, although the results were discordant between the COI and internal transcribed spacer (ITS) [79]. In Madagascar, ITS2 was also insufficient to resolve the species and species complexes in the *Neomelaniconion* subgenus [80]. Further studies using different markers in combination with morphology are needed to resolve uncertainties in the *Neomelaniconion* subgenus.

*Aedes cumminsii* collected in South Africa did not cluster together with sequences from specimens identified as this species in Kenya, Guinea, and Senegal. This species was originally described in Ghana and is widely distributed in Africa. Subspecies based on subtle differences in abdomen scaling have been described, such as ssp. *mediopunctatus* (Theobald) [18], and it is not surprising that specimens from South Africa are different. *Aedes cumminsii* likely represents a complex of species which will require further studies to elucidate their taxonomy and importance as vectors of SPOV, MIDV, SINV, and RVF [57, 81–83]. McIntosh [56] suggested that only ssp. *mediopunctatus* occurs in South Africa. However, Jupp [20] used morphological characters from *mediopunctatus* in his key, naming the species *cumminsii*. Morphologically, species analysed in this study had a proboscis entirely dark-scaled, scutum indistinctly marked, scutellum with narrow yellow scales, hind tarsal ungues armed, large mosquito with wing length about 5.0 mm, in agreement with descriptions for the typical *Ae. cumminsii* species. However, they also had a long medium basal band on the abdominal tergites, which according to Edwards [18] is more characteristic of ssp. *mediopunctatus*. Therefore, the results presented here suggest that ssp. *mediopunctatus* occurs in north-eastern areas of South Africa. Further studies would be necessary to clarify the status of *Ae. cumminsii* in South Africa.

## Conclusions

There is a paucity of information on *Aedes* species ecology across southern Africa. Because of the importance of Aedes as disease vectors and the expansion of arboviruses globally over the past two decades due to the increased travel, trade, and climate change, investigations are urgently needed to update the records of sylvatic species. Although the survey area was limited geographically to the north-eastern parts of South Africa, and despite its limited overall trap coverage, the study provides a better understanding of the diversity and relative abundance of *Aedes* species in these areas where arboviruses are known to be active.

The most abundant aedine species caught were those belonging to the *Neomelaniconion* subgenus, which are recognised as potential arboviral vectors, especially *Ae. mcintoshi*, enabling the propagation of arboviral infection in animals and humans in the study area. *Aedes aegypti* was present in almost all sites sampled, predominantly in urban areas, albeit in low numbers. This study also provided initial data on the feeding behaviour of *Aedes* mosquitoes in the collection sites, reflecting potential reservoirs for the viruses they transmit in Africa and risk of transmission to human and domestic species. We provided information about the feeding behaviour of sylvatic species including *Ae. eritreae/karooensis*, *Ae. leesoni/alboventralis*, *Ae. pachyurus*, and *Ae. aurovenatus*, which was not previously available in the literature. The results presented here show that molecular characterisation was able to be successfully employed for species identification of *Aedes* mosquitoes in South Africa, and contribute to expanding the DNA barcode references available for African *Aedes*. Apart from confirming the presence and genetic data of several common vector species across the study region, this study added COI sequences for nine additional species to the open-access online databases, namely *Ae. eritreae*, *Ae. pachyurus*, *Ae. ledgeri*, *Ae. juppi*, *Ae. microstictus*, *Ae. aurovenatus*, *Ae. unidentatus*, *Ae. durbanensis*, and *Ae. haworthi*.

## Supplementary Information

**Additional file 1**: **Table S1. **Collection sites for mosquito samples surveyed from January 2014 to May 2018. **Table S2. **Number of *Aedes *collected at sentinel sites, relative abundance (%), and diversity indexes, January 2014 to June 2018. **Table S3. **Number of *Aedes *collected at ad hoc sites, relative abundance (%), and diversity indexes, January 2014 to June 2018. **Table S4. **Comparison of the abundance of *Aedes *captured by the three trap types at the sentinel sites from 2017 and 2018. Table showing the results of the Kruskal–Wallis multiple tests. **Table S5. **Correlation coefficient between *Aedes *per trap-night and the environmental conditions. **Table S6. ***Aedes *blood meals identified in various mammal and avian species. **Figure S1. **Relative abundance (%) of *Aedes *vectors per trap-night from January 2014 to June 2018 at the sentinel sites. **Figure S2. **Most common potential *Aedes *vectors abundance per trap-night, rainfall, and temperatures from January 2014 to June 2018 at the sentinel sites. **Figure S3. **Regression relationship between log10 of the *Aedes *per trap-night and average temperature 30 and 15 days prior to collection, average rainfall 30 and 15 days prior to the collection event, and average humidity 30 and 15 days prior to collection, January 2014 to June 2018. **Figure S4. **The morphological characteristics of an adult female of *Ae. cumminsii *collected in South Africa. A) Dorsal view; B) Lateral view.

## Data Availability

All data generated or analysed during this study are included in this published article and its Additional files.

## Abbreviations

BG: Biogents
CHIKV: Chikungunya virus
CO_2_: Carbon dioxide
COI: Cytochrome oxidase subunit I
CDC: Centers for Disease Control and Prevention
DENV: Dengue virus
ITS: Internal transcribed spacer
KNP: Kruger National Park
K2P: Kimura 2-parameter model
MAFFT: Multiple alignment using fast Fourier transform
MEGA: Molecular evolutionary genetics analysis
MIDV: Middelburg virus
NICD: National Institute for Communicable Diseases of South Africa
RVF: Rift Valley fever
SAWS: South African Weather Service
SINV: Sindbis virus
SHOV: Shokwe virus
SPOV: Spondweni virus
spp: Species
ssp: Subspecies
WSLB: Wesselsbron virus
YFV: Yellow fever virus
ZIKV: Zika virus
