# Supplementary material for: Aedes species (Diptera: Culicidae) ecological and host feeding patterns in the north-eastern parts of South Africa, 2014–2018
Source: Parasit Vectors. 2021 Jun 26;14:339. doi: 10.1186/s13071-021-04845-9 (PMC8235819; doi:10.1186/s13071-021-04845-9)

Additional File 1

Table 1: Collection sites for mosquito samples surveyed from January 2014 to May 2018.

Site	Coordinates		Land-use	Animals Present
Pretoria North	-25,68663	28,15895	Urban	Humans, birds, and dogs
Matikwane	-24.985447	31.236342	Urban	Humans, birds, and dogs
Boshckop	-25,82786	28,42047	Horse Farm	Horses, humans, goats, sheep, rabbits, and mongooses
Kyalami	-25,99183	28,02947	Horse Farm	Horses, humans, goats, sheep, and Egyptian geese
Benoni	-26,10611	28,36689	Horse Farm	Humans, horses, and dogs
Roodeplaat	-25,62075	28,37136	Peri urban	Humans, birds, dogs, and impalas
Vulpro	-25,7112	27,95322	Vulture rehabilitation Centre	Vultures, Horses, dogs, and humans
Mnisi	-24,48206	31,38583	Rural, border Knp	Humans, dogs, pigs, goats, cattle, donkeys, and birds
Jozini	-27,41258	32,20647	Rural	Humans, dogs, pigs, goats, cattle, and donkeys
Hectorspruit	-25.448702	31.70438	Rural, border Knp	Humans, dogs, pigs, goats, cattle, and donkeys
Cork	-24.954514	31.300033	Rural, border Knp	Humans, dogs, pigs, goats, cattle, and donkeys
Maluleke	-22.865464	30.929738	Rural, border Knp	Humans, dogs, pigs, goats, cattle, and donkeys
Welverdiend	-24.59252	31.348995	Rural, border Knp	Humans, dogs, pigs, goats, cattle, and donkeys
Marakele	-24,29364	27,50325	Wildlife Reserve	Wildlife, birds, and humans
Lapalala	-23,88458	28,26953	Wildlife Reserve	Wildlife, birds, and humans
KNP Shingwedzi	-23,10819	31,43628	Wildlife Reserve	Wildlife, birds, and humans
KNP Skukukuza	-24,99633	31,59189	Wildlife Reserve	Wildlife, birds, and humans
KNP Satara	-25.35384	31.79936	Wildlife Reserve	Wildlife, birds, and humans
KNP Malelane	-25.37349	31.60691	Wildlife Reserve	Wildlife, birds, and humans
KNP Punda Maria	-22.756171	31.009136	Wildlife Reserve	Wildlife, birds, and humans

KNP: Kruger National Park

Table 2: Number of *Aedes* collected at sentinel sites, relative abundance (%) and diversity indexes, January 2014 to June 2018.

	Sentinel Sites							
	Conservation Areas				Peri Urban			
**Sites**	**Marakele**		**Lapalala**		**Kyalami**		**Boschkop**	
**Species**	N	%	N	%	N	%	N	%
Ae (Muc) sudanensis	13	0.44	6	0.24	1	0.17	1	0.15
Ae (Aed) vittatus	17	0.58	544	21.57	2	0.35	2	0.30
Ae (Aed) vexans	3	0.10	0	0.00	0	0.00	0	0.00
Ae (Aed) hirsutus	117	3.98	61	2.42	5	0.87	4	0.60
Ae (Aed) fowleri	29	0.99	99	3.93	6	1.04	1	0.15
Ae (Ae) durbanensis	0	0.00	0	0.00	0	0.00	1	0.15
Ae (Aed) vexans group	0	0.00	3	0.12	1	0.17	2	0.30
Ae (Aed) cumminsii	18	0.61	5	0.20	1	0.17	5	0.75
Ae (Aed) pachyurus	0	0.00	0	0.00	40	6.93	43	6.48
Ae (Aed) dentatus group	0	0.00	0	0.00	19	3.29	98	14.76
Ae. (Aed) leesoni group	0	0.00	0	0.00	93	16.12	56	8.43
Ae (Aed) dentatus	0	0.00	0	0.00	117	20.28	0	0.00
Ae (Aed) eritreae	0	0.00	0	0.00	0	0.00	4	0.60
Ae (Aed) ochraceus	16	0.54	4	0.16	0	0.00	0	0.00
Ae (Aed) argenteopunctatus	13	0.44	8	0.32	2	0.35	0	0.00
Ae (Aed) microstictus	5	0.17	65	2.58	0	0.00	0	0.00
Ae (Aed) bedfordi	0	0.00	0	0.00	0	0.00	0	0.00
Ae (Aed) filicis	0	0.00	0	0.00	0	0.00	0	0.00
Ae (Aed) quasiunivittatus	11	0.37	322	12.77	31	5.37	276	41.57
Ae (Aed) spp.	0	0.00	1	0.04	83	14.38	13	1.96
Ae (Alb) marshalis	0	0.00	2	0.08	0	0.00	0	0.00
Ae (Alb) haworthi	0	0.00	0	0.00	0	0.00	0	0.00
Ae (Och) juppi	2	0.07	0	0.00	19	3.29	33	4.97
Ae (Och) caballus	2	0.07	0	0.00	0	0.00	0	0.00
Ae (Och) breedensis	1	0.03	0	0.00	0	0.00	0	0.00
Ae (Ste) aegypti	17	0.58	15	0.59	60	10.40	50	7.53
Ae (Ste) unilineatus	3	0.10	26	1.03	0	0.00	0	0.00
Ae (Ste) metallicus	7	0.24	21	0.83	1	0.17	2	0.30
Ae (Ste) heishi	0	0.00	1	0.04	0	0.00	0	0.00
Ae (Ste) ledgeri	0	0.00	13	0.52	0	0.00	0	0.00
Ae (Ste) subargenteus	0	0.00	0	0.00	0	0.00	0	0.00
Ae (Ste) simpsoni	0	0.00	0	0.00	2	0.35	4	0.60
Ae (Ste) spp.	0	0.00	27	1.07	6	1.04	4	0.60
Ae (Dic) furcifer/cordellieri	1	0.03	5	0.20	0	0.00	0	0.00
Ae (Dic) fascipalpis	1	0.03	1	0.04	0	0.00	0	0.00
Ae (Neo) mcintoshi	2434	82.82	1075	42.62	17	2.95	29	4.37
Ae (Neo) unidentatus	0	0.00	2	0.08	18	3.12	3	0.45
Ae (Neo) circumluteolus	154	5.24	34	1.35	0	0.00	0	0.00
Ae (Neo) luteolateralis	0	0.00	0	0.00	0	0.00	0	0.00
Ae (Neo) albothorax	2	0.07	5	0.20	0	0.00	0	0.00
Ae (Aed) aerarius	0	0.00	6	0.24	0	0.00	0	0.00
Ae (Aed) veeniae	0	0.00	0	0.00	0	0.00	0	0.00
Aedes spp.	73	2.48	171	6.78	53	9.19	33	4.97
**Total**	2939		2522		577		664	
**Taxa_S**	22		26		21		21	
**Simpson_1-D**	0,3089		0,7475		0,8809		0,781	

Table 3: Number of *Aedes* collected at *ad-hoc* sites, relative abundance (%) and diversity indexes, January 2014 to June 2018.

	***Ad-Hoc* Sites**																	
	**Rural**				**Conservation Areas**				**Urban**				**Peri Urban**					
**Sites**	**Mnisi**		**Jozini**		**KNP Shingwedzi**		**KNP Skukuza**		**Pretoria North**		**Matikwane**		**Benoni**		**Roodeplaat**		**Vulpro**	
**Species**	N	%	N	%	N	%	N	%	N	%	N	%	N	%	N	%	N	%
Ae (Muc) sudanensis	3	0.35	12	0.51	1	1.27	1	2.00	0	0.00	0	0.00	0	0.00	1	0.97	0	0.00
Ae (Aed) vittatus	72	8.33	0	0.00	3	3.80	0	0.00	0	0.00	2	0.58	0	0.00	0	0.00	0	0.00
Ae (Aed) vexans	1	0.12	5	0.21	0	0.00	0	0.00	0	0.00	0	0.00	0	0.00	0	0.00	0	0.00
Ae (Aed) hirsutus	13	1.50	13	0.55	7	8.86	3	6.00	0	0.00	1	0.29	0	0.00	0	0.00	9	2.63
Ae (Aed) fowleri	7	0.81	0	0.00	0	0.00	0	0.00	0	0.00	0	0.00	0	0.00	12	11.65	0	0.00
Ae (Ae) durbanensis	0	0.00	1556	66.27	1	1.27	0	0.00	0	0.00	0	0.00	0	0.00	0	0.00	0	0.00
Ae (Aed) vexans group	0	0.00	0	0.00	0	0.00	0	0.00	0	0.00	0	0.00	0	0.00	2	1.94	0	0.00
Ae (Aed) cumminsii	0	0.00	50	2.13	7	8.86	1	2.00	0	0.00	0	0.00	0	0.00	0	0.00	4	1.17
Ae (Aed) pachyurus	0	0.00	0	0.00	0	0.00	1	2.00	0	0.00	0	0.00	29	8.33	18	17.48	0	0.00
Ae (Aed) dentatus group	0	0.00	1	0.04	1	1.27	0	0.00	0	0.00	0	0.00	233	66.95	1	0.97	0	0.00
Ae. (Aed) leesoni group	2	0.23	0	0.00	0	0.00	0	0.00	0	0.00	0	0.00	1	0.29	0	0.00	0	0.00
Ae (Aed) dentatus	0	0.00	0	0.00	0	0.00	0	0.00	0	0.00	0	0.00	0	0.00	0	0.00	0	0.00
Ae (Aed) eritreae	0	0.00	0	0.00	0	0.00	0	0.00	0	0.00	0	0.00	0	0.00	0	0.00	159	46.49
Ae (Aed) ochraceus	0	0.00	13	0.55	23	29.11	2	4.00	0	0.00	0	0.00	0	0.00	0	0.00	0	0.00
Ae (Aed) argenteopunctatus	0	0.00	0	0.00	0	0.00	0	0.00	0	0.00	0	0.00	3	0.86	4	3.88	0	0.00
Ae (Aed) microstictus	0	0.00	0	0.00	0	0.00	0	0.00	0	0.00	0	0.00	0	0.00	0	0.00	0	0.00
Ae (Aed) bedfordi	0	0.00	0	0.00	4	5.06	2	4.00	0	0.00	0	0.00	0	0.00	0	0.00	0	0.00
Ae (Aed) filicis	7	0.81	0	0.00	0	0.00	0	0.00	0	0.00	0	0.00	0	0.00	0	0.00	0	0.00
Ae (Aed) quasiunivittatus	12	1.39	0	0.00	0	0.00	0	0.00	0	0.00	0	0.00	0	0.00	0	0.00	0	0.00
Ae (Aed) spp.	7	0.81	12	0.51	0	0.00	1	2.00	0	0.00	1	0.29	4	1.15	4	3.88	0	0.00

Ae (Alb) marshalis	0	0.00	0	0.00	0	0.00	0	0.00	0	0.00	0	0.00	0	0.00	0	0.00	0	0.00
Ae (Alb) haworthi	2	0.23	0	0.00	0	0.00	0	0.00	0	0.00	0	0.00	0	0.00	0	0.00	0	0.00
Ae (Och) juppi	0	0.00	0	0.00	0	0.00	0	0.00	0	0.00	0	0.00	0	0.00	0	0.00	25	7.31
Ae (Och) caballus	0	0.00	0	0.00	0	0.00	0	0.00	0	0.00	0	0.00	0	0.00	0	0.00	0	0.00
Ae (Och) breedensis	0	0.00	0	0.00	0	0.00	0	0.00	0	0.00	0	0.00	0	0.00	0	0.00	0	0.00
Ae (Ste) aegypti	206	23.84	26	1.11	3	3.80	1	2.00	209	96.31	278	80.58	4	1.15	2	1.94	12	3.51
Ae (Ste) unilineatus	23	2.66	0	0.00	2	2.53	0	0.00	0	0.00	0	0.00	0	0.00	0	0.00	0	0.00
Ae (Ste) metallicus	15	1.74	10	0.43	3	3.80	0	0.00	0	0.00	0	0.00	1	0.29	0	0.00	0	0.00
Ae (Ste) heishi	0	0.00	0	0.00	0	0.00	0	0.00	0	0.00	0	0.00	0	0.00	0	0.00	0	0.00
Ae (Ste) ledgeri	0	0.00	2	0.09	0	0.00	0	0.00	3	1.38	0	0.00	0	0.00	0	0.00	0	0.00
Ae (Ste) subargenteus	0	0.00	8	0.34	0	0.00	0	0.00	0	0.00	0	0.00	0	0.00	0	0.00	0	0.00
Ae (Ste) simpsoni	0	0.00	0	0.00	0	0.00	0	0.00	5	2.30	58	16.81	0	0.00	0	0.00	0	0.00
Ae (Ste) spp.	4	0.46	26	1.11	1	1.27	1	2.00	0	0.00	3	0.87	1	0.29	0	0.00	0	0.00
Ae (Dic) furcifer/cordellieri	1	0.12	0	0.00	0	0.00	0	0.00	0	0.00	0	0.00	0	0.00	0	0.00	0	0.00
Ae (Dic) fascipalpis	1	0.12	0	0.00	0	0.00	0	0.00	0	0.00	0	0.00	0	0.00	0	0.00	0	0.00
Ae (Neo) mcintoshi	410	47.45	26	1.11	21	26.58	8	16.00	0	0.00	1	0.29	7	2.01	57	55.34	133	38.89
Ae (Neo) unidentatus	0	0.00	0	0.00	0	0.00	0	0.00	0	0.00	0	0.00	50	14.37	2	1.94	0	0.00
Ae (Neo) circumluteolus	0	0.00	480	20.44	1	1.27	28	56.00	0	0.00	1	0.29	4	1.15	0	0.00	0	0.00
Ae (Neo) luteolateralis	1	0.12	0	0.00	0	0.00	0	0.00	0	0.00	0	0.00	0	0.00	0	0.00	0	0.00
Ae (Neo) albothorax	0	0.00	0	0.00	0	0.00	0	0.00	0	0.00	0	0.00	0	0.00	0	0.00	0	0.00
Ae (Aed) aerarius	1	0.12	0	0.00	0	0.00	0	0.00	0	0.00	0	0.00	1	0.29	0	0.00	0	0.00
Ae (Aed) veeniae	0	0.00	3	0.13	0	0.00	0	0.00	0	0.00	0	0.00	0	0.00	0	0.00	0	0.00
Aedes spp.	76	8.80	105	4.47	1	1.27	1	2.00	0	0.00	0	0.00	10	2.87	0	0.00	0	0.00
**Total**	864		2348		79		50		217		345		348		103		342	
**Taxa_S**	20		17		15		12		3		8		13		10		6	
**Simpson_1-D**	0.702		0.516		0.82		0.651		0.072		0.322		0.522		0.645		0.625	

Table 4: Comparison of the abundance of *Aedes* captured by the three trap types at the sentinel sites from 2017 and 2018. Table showing the results of the Kruskal-Wallis multiple tests.

Trap comparison	Test Statistic	Std. Error	Std. Test Statistic	Sig.	Adj. Sig.*
BG-MTT	31.067	13.723	2.264	0.024	0.071
BG-CDC	58.358	13.723	4.252	0	0
MTT-CDC	-27.292	13.723	-1.989	0.047	0.14

* Significance values have been adjusted by the Bonferroni correction for multiple tests. The significance level is 0,05

Table 5: Correlation coefficient between *Aedes* per trap-night and the environmental conditions.

Variable	Correlation Coeficient (r)	Sig (2 tailed)	N
Temperature 15 days	0,496	0	292
Temperature 30 days	0,533	0	292
Temperature daily	0,415	0	287
Temperature nightly	0,408	0	288
Temperature 48h	0,429	0	288
Rainfall nightly	0,179	0.002	292
Rainfall 48h	0,263	0	292
Rainfall 15 days	0,375	0	292
Rainfall 30 days	0,498	0	292
Humidity daily	0,264	0	265
Humidity nightly	0,280	0	265
Humidity 15 days	0,388	0	272
Humidity 30 days	0,384	0	278
Humidity 48h	0,325	0	265
Wind daily	-0,225	0	278
Wind nightly	-0,149	0.013	279
Wind 48h	-0,138	0.021	279

Rainfall daily	0.041	0.492	288

*Correlation is significant at the 0.05 level (2-tailed).

Table 6: *Aedes* blood meals identified in various mammal and avian species.

Sites	No. Positive mammals	No. Positive avians	Pools tested	*Syncerus caffer*	*Connochaetes taurinus*	*Sylvicapra grimmia*	*Tragelaphus scriptus*	*Felis silvestris catus*	*Bos taurus*	*Capra hircus*	*Hippopotamus amphibious*	*Equus caballus*	*Homo sapiens*	*Aepyceros melampus*	*Tragelaphus strepsiceros*	*Tragelaphus angasii*	*Ovies aries*	*Gyps coprotheres*	*Kobus ellipsiprymnus*	*Ceratotherium simum*	*Dryocopus gambensis*	*Canis lupus*
**Benoni**	4	0	9	-	-	-	-	-	2	-	-	-	1	-	-	-	-	-	-	-	-	1*
**Boschkop**	1	-	1	-	-	-	-	-	-	-	-	-	-	-	-	-	1	-	-	-	-	-
**Kyalami**	3	0	8	-	-	-	-	1	-	1	-	1	-	-	-	-	-	-	-	-	-	-
**Vulpro**	6	1	9	-	1	-	1	-	-	-	-	-	3	-	-	1	-	1	-	-	-	-
**Roodeplaat**	2	-	2	-	-	1	-	-	-	-	-	-	-	-	-	1*	-	-	-	-	-	-
**Marakele**	11	0	19	1	1	-	1*	-	-	-	-	-	3	2	2, 1*	-	-	-	-	-	-	-
**Lapalala**	6	0	18	-	-	-	-	-	-	-	2	-	1	-	-	-	-	-	1	2	-	-
**Mnisi**	4	0	6	-	-	-	-	-	3	-	-	-	-	1	-	-	-	-	-	-	-	-
**KNP-SHI**	13	1	23	10	-	-	1	-	-	-	-	-	-	1	1	-	-	-	-	-	1*	-
**KNP-Mal**	2	0	3	-	-	-	-	-	-	-	-	-	-	-	-	-	-	-	-	2	-	-
**KNP-Sat**	3	0	4	-	-	-	-	-	-	-	-	-	-	3	-	-	-	-	-	-	-	-
**Jozini**	7	0	10	-	-	-	-	-	4	1	-	-	-	-	-	-	2	-	-	-	-	-
**Total**	**62**	**2**	**112**	**11**	**2**	**2**	**1**	**1**	**9**	**2**	**2**	**1**	**8**	**7**	**1**	**1**	**3**	**1**	**1**	**4**	**1**	**1**
**Percentage**	**55.36**	**1.79**	**-**	**17**	**3.1**	**3.1**	**1.6**	**1.6**	**14**	**3.1**	**3.1**	**1.6**	**13**	**11**	**1.6**	**1.6**	**4.7**	**1.6**	**1.6**	**6.3**	**1.6**	**1.6**

***** BLAST sequences less than 97.0%.

Figure 1: Relative abundance (%) of *Aedes* vectors abundance per trap-night from January 2014 to June 2018 at the sentinel sites.

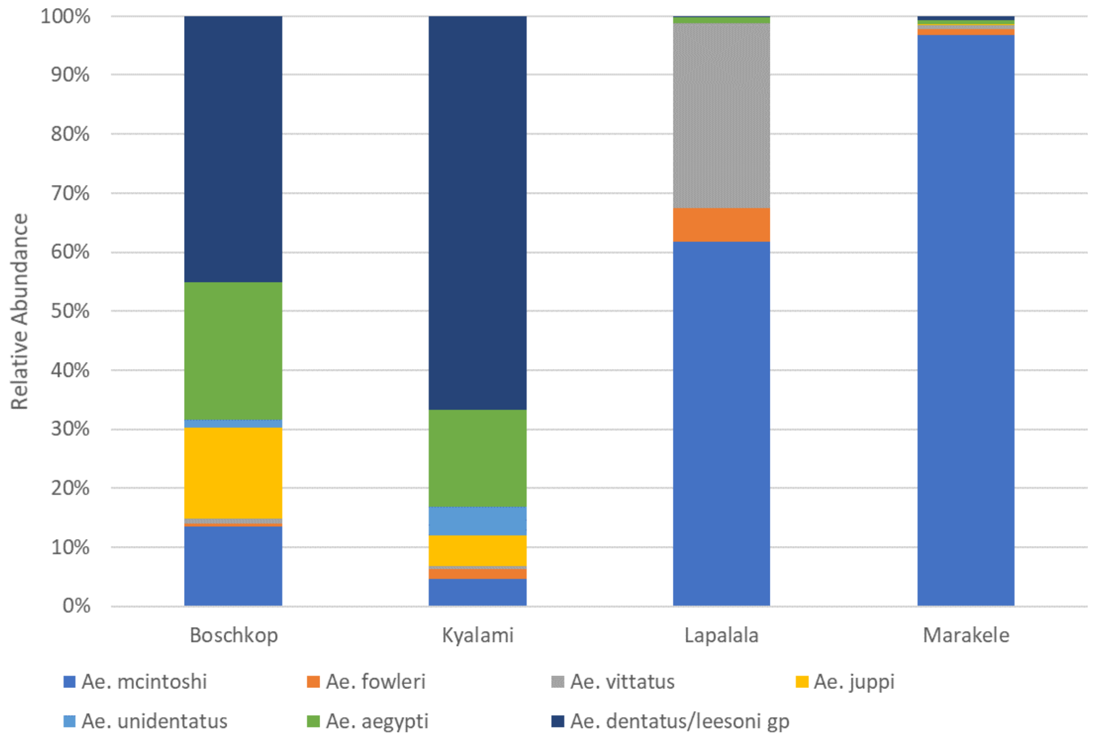

Figure 2: Most common potential *Aedes* vectors abundance per trap-night, rainfall, and temperature from January 2014 to June 2018 at the sentinel sites.

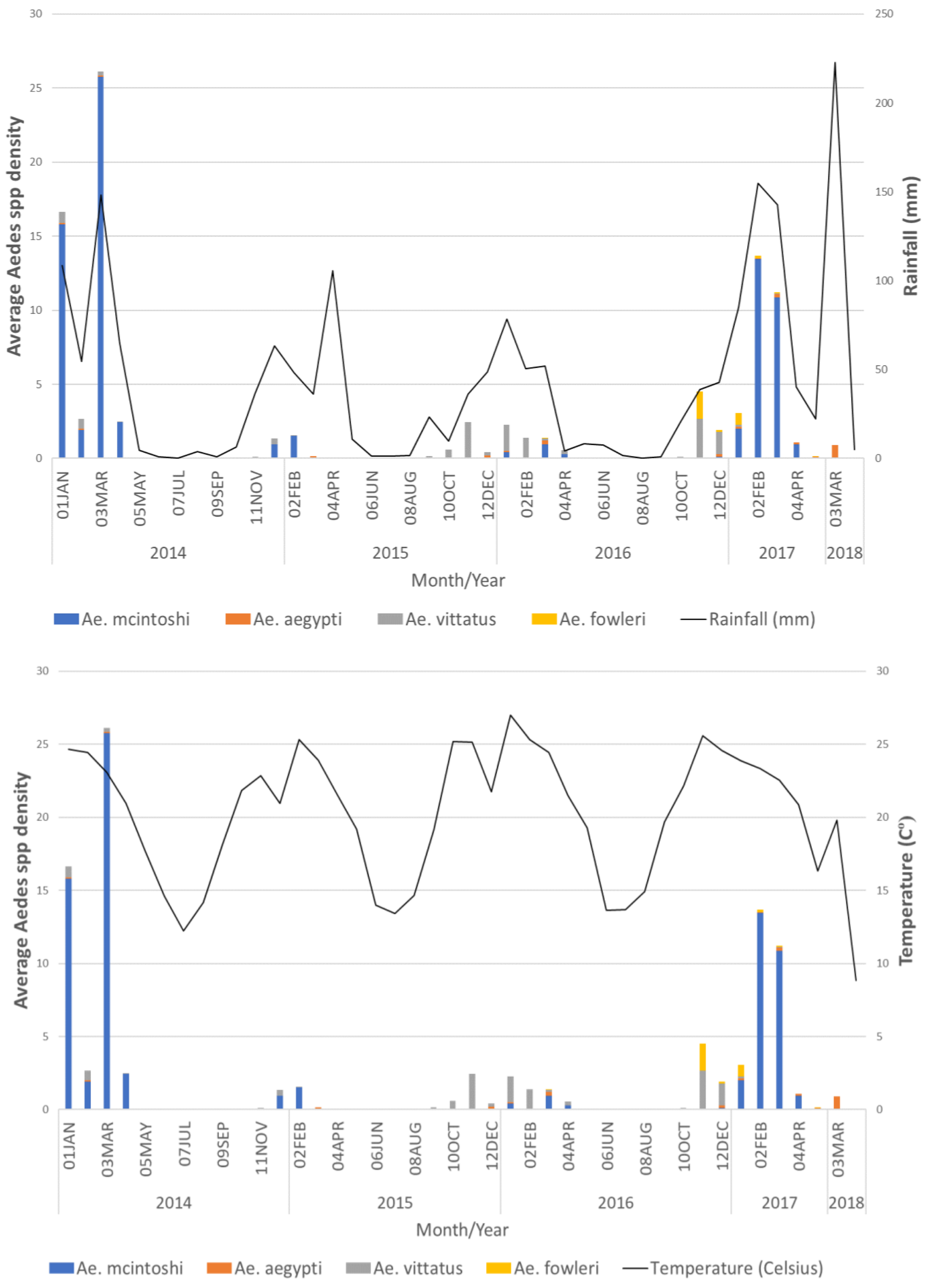

Figure 3: Regression relationship between $\log_{10}$ of the *Aedes* per trap-night and average of temperature 30 and 15 days prior to collection, average of rainfall 30 and 15 days prior to the collection event, and average of humidity 30 and 15 days prior to collection, January 2014 to June 2018.

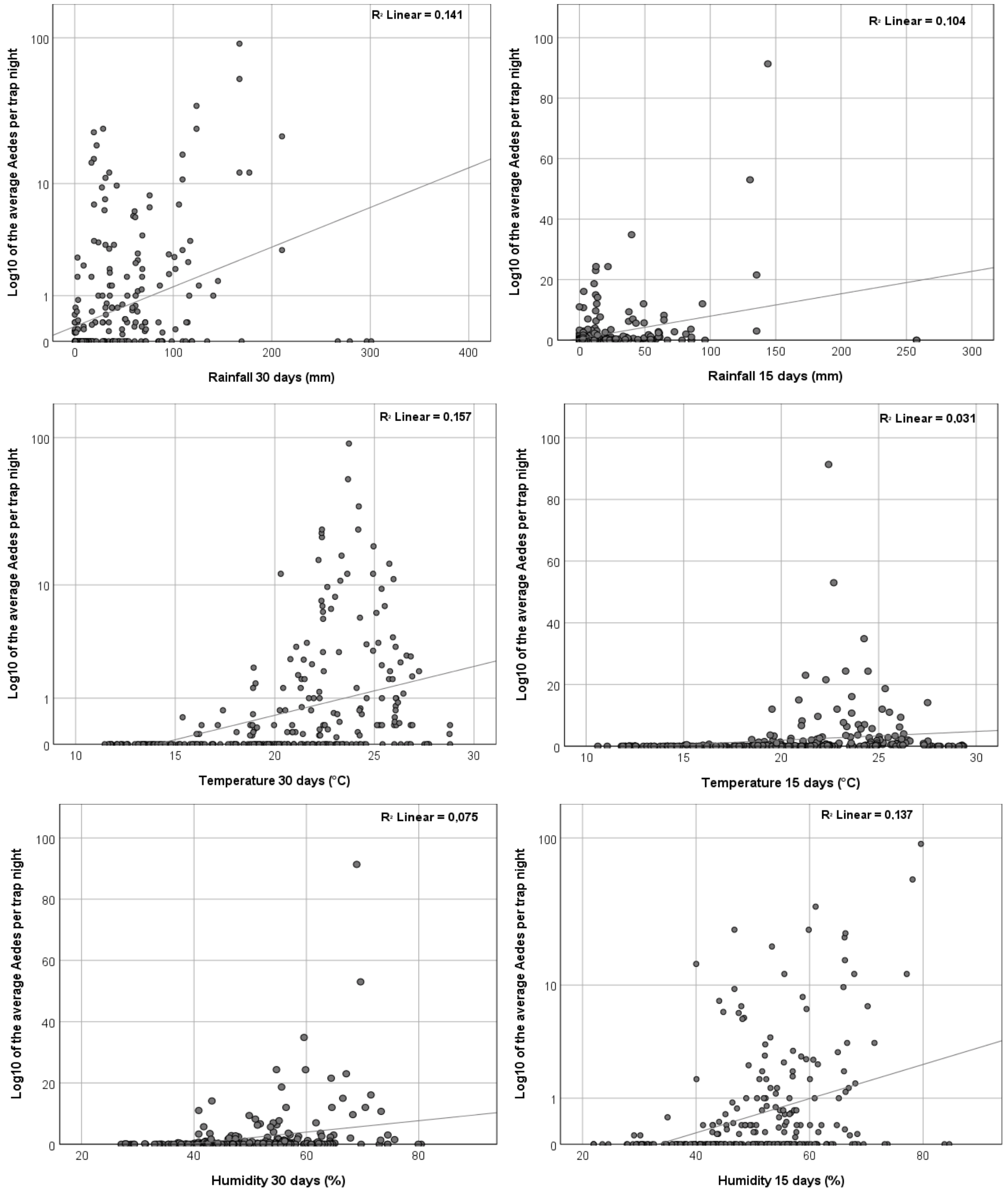

Figure 4: Represents the morphological characteristics of an adult female of *Ae. cumminsii* collected in South Africa. A) Dorsal view; B) Lateral view.

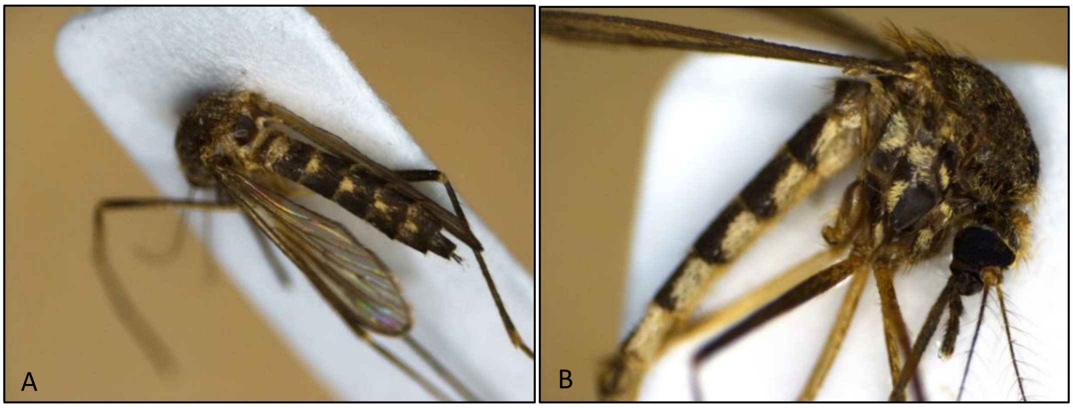

Supplement: Supplementary file 1 — Additional file 1: Table S1. Collection sites for mosquito samples surveyed from January 2014 to May 2018. Table S2. Number of Aedes collected at sentinel sites, relative abundance (%), and diversity indexes, January 2014 to June 2018. Table S3. Number of Aedes collected at ad hoc sites, relative abundance (%), and diversity indexes, January 2014 to June 2018. Table S4. Comparison of the abundance of Aedes captured by the three trap types at the sentinel sites from 2017 and 2018. Table showing the results of the Kruskal–Wallis multiple tests. Table S5. Correlation coefficient between Aedes per trap-night and the environmental conditions. Table S6. Aedes blood meals identified in various mammal and avian species. Figure S1. Relative abundance (%) of Aedes vectors per trap-night from January 2014 to June 2018 at the sentinel sites. Figure S2. Most common potential Aedes vectors abundance per trap-night, rainfall, and temperatures from January 2014 to June 2018 at the sentinel sites. Figure S3. Regression relationship between log10 of the Aedes per trap-night and average temperature 30 and 15 days prior to collection, average rainfall 30 and 15 days prior to the collection event, and average humidity 30 and 15 days prior to collection, January 2014 to June 2018. Figure S4. The morphological characteristics of an adult female of Ae. cumminsii collected in South Africa. A) Dorsal view; B) Lateral view. [file 13071_2021_4845_MOESM1_ESM.pdf]
